# Design and simulation of a highly efficient eco-friendly, non-toxic perovskite solar cell

**DOI:** 10.1186/s11671-025-04190-1

**Published:** 2025-02-12

**Authors:** G. S. Ahathiyan, H. Victor Du John, D. Jackuline Moni, K. Martin Sagayam, Binay Kumar Pandey, Digvijay Pandey, Mesfin Esayas Lelisho

**Affiliations:** 1 Division of Electronics and Communication Engineering, Karunya Institute of Technology and Sciences, Coimbatore, India; 2 Department of Information Technology, College of Technology, Govind Ballabh Pant University of Agriculture and Technology, Pantnagar, Uttrarakhand India; 3 Department of Technical Education Uttar Pradesh, Kanpur, India; 4 Department of Statistics, College of Natural and Computational Science, Mizan-Tepi University, Tepi, Ethiopia

**Keywords:** Non-toxic, Perovskite solar cell, SCAPS-1D, Eco-friendly, Lead-free

## Abstract

A highly efficient and nontoxic material methylammoniumtin(II) iodideperovskite solar cell is proposed. This proposed solar cell uses CH_3_NH_3_SnI_3_ as the absorber layer, TiO_2_ as an Electron transport layer (ETL), Indium tin oxide as a buffer layer, and Copper(I) oxide as the hole transport layer (HTL). The device is simulated using the SCAPS-1D simulation tool. This study details the optimization of a set of parameters, including the defect densities and the thickness of the absorber layer. The proposed structure is highly optimized result of 31.73% of enhanced power conversion efficiency (PCE), a J_SC_ of 24.526 mA/cm^2^ (short-circuit current), FF of 81.40% (fill factor), and a V_OC_ of 1.56 V (open-circuit voltage) is obtained through simulation process. Compared to previously reported works, the performance of the device has improved significantly due to better optimization. Along with this electrical characteristic temperature analyses, conductance voltage, capacitance–voltage, and bandgap analyses have also been carried out to examine the device’s efficiency and performance.

## Introduction

Day by day the usage of energy is becoming higher. Most of the energy we consume is from coal, oil, and natural gas, which are non-renewable. This also causes pollution and leads to global warming [1]. By overconsuming this type of energy at a certain time, we run out of energy in a very short period. Further use of these fossil fuels results in significant impacts to the environment and climate, which also affects the ecosystem [2]. To avoid this situation the solution is to move to renewable resources like solar energy, windmills, etc. One of the alternate solutions is (renewable resource) solar cells. Compared with other renewable energy sources one of the best options or alternatives is solar energy because of its abundance in nature, freely available and we can use this energy most of the time. Because of the ozone layer and clouds, which reflect, absorb, and disperse light, the majority of solar rays are wasted [3]. Normally, the sun emits solar radiation of 3.8 × 1023 kW, out of which the earth’s surface receives approximately 1.8 × 1014 kW, we can use this energy without altering its state [4]. Using this, we can get sufficient energy without polluting the environment. The most widely used energy is thermal energy and the least energy we use is solar energy.

Solar cells come in diverse varieties and have four generations. First-generation solar cells are mainly composed of crystalline silicon and are expensive yet very efficient. To extract energy from photons, they employ a single junction and get close to the maximum theoretical efficiency of 33%. Although first-generation cells are widely used, their manufacturing is intrinsically costly. In comparison to first generation solar cells second generation solar cells are less expensive and less efficient, which are often referred to as thin-film solar cells, their production methods are low-cost and need few components. Materials consist of silicon, amorphous silicon, CdTe, and CIGS. Although they have a lower conversion rate (between 10 and 15%), the lower cost offsets this disadvantage. Third-generation cells have not yet reached full commercialization and are still in the research stage. The objective is to create inexpensive, incredibly effective cells. New materials and design strategies strive for higher efficiency. Third-generation cell technologies include tandem cells, perovskite cells, multijunction photovoltaic cells, and nanostructured cells [5]. Fourth-generation solar Cells, or “inorganics-in-organics,” integrate the greatest aspects of both worlds. They combine the robustness of cutting-edge inorganic nanostructures with the affordability and pliability of polymer thin films. Organic-based nanomaterials (such as graphene and its derivatives) contain embedded inorganic nanostructures (metal nanoparticles or metal oxides) [5, 6]. The goals of fourth-generation cells include great cost-effectiveness, durability, and efficiency.

A (PSC) perovskite solar cell is a solar cell that uses a perovskite-structured material as the light-harvesting active layer which can produce electricity. Relative to conventional solar panels, perovskite solar cells function similarly. These compounds are usually lead or tin halides are hybrid organic–inorganic compounds used as active layers in perovskite solar cells. Methyl ammonium lead halides and all-inorganic cesium lead halides are two examples of perovskite materials that are inexpensive and easy to make. The rapid improvement of perovskite’s performance efficiency has made them a game-changer in the field of photovoltaics. When considering perovskite in comparison to traditional silicon-based solar cells, two important benefits are their high tunability and inexpensive fabrication costs. Over the last decade, their efficiency has improved from 3.5 to 25.8% [7]. Due to their promise for flexible, low-cost energy conversion and their notable efficiency increases, which drawn a lot of attention.

Lev Perovski, a Russian mineralogist, gave the material its name after it was initially for by Gustavo Rose in Russia in the year 1839. Perovskite solar cells are a particular type of solar cell with a perovskite structural component. The absorber material ABX₃ crystal structure is where the term “perovskite solar cell” originated. A and B are two cations in ABX_3_ crystal structure, both A and B bond to X anion. The ABX_3_ is a crystal structure, which is an absorber material, which is used to create the perovskite solar cell. Perovskite solar cells have a sandwich-like structure. As the light-harvesting active layer, hybrid organic–inorganic metal halide materials are most frequently used. In our device, we have used a CH_3_NH_3_SnI_3_-based perovskite material which is a lead-free material in nature. Rose discovered perovskite structure material in Miyasaka in 2009, and she used this specific material [8, 9] in the solar cell. Some advantages of perovskite solar cells are producing perovskite materials is cheap, and cost-effectiveness is enhanced by production processes like printing, and perovskite sheets, which are incredibly thin (about cells can absorb a wide variety of photon energy).The following graph shows the emerging PV cell efficiency from NREL [10].

In the domain of solar cell simulation software, the creation and simulation of a perovskite solar cell in SCAPS-1D were undertaken [11]. This endeavour was spearheaded by the ELIS Department at Gent University in Belgium, resulting in the development of the Solar Cell-Capacitance Simulator application. SCAPS, serving as a one-dimensional simulator tool for solar cells, was employed for this purpose.

## Related work

To study the electrical and optical characteristics of solar cells, SCAPS-1D (Open-Source) is a simulation tool is used. The tool makes it possible to model the electrical properties of solar cells, examine the impacts of various layouts and materials, and adjust device settings to increase efficiency. SCAPS-1D optimizes the thickness of various layers, series, and shunt resistances, and temperature to improve the performance of perovskite solar cells [12]. This tool can simulate a broad variety of materials, including single-walled carbon nanotubes, organic compounds, and lead-free perovskites, allowing customized designs for specific applications [13]. To maximize power conversion efficiency, the tool makes it easier to optimize doping concentrations, defect density, and layer thickness [14].

Perovskite solar cells (PSCs) have emerged as a viable photovoltaic technology owing to its high power conversion efficiency (PCE) and low-cost manufacturing techniques. A variety of PSCs have been produced, each having its structure and materials to improve performance and stability. Solid-state PSCs are an excellent choice for next-generation solar energy harvesters because of their high efficiency and low-cost materials/processes [15]. Originally evolved from dye-sensitized solar cells, these cells encountered stability challenges owing to perovskite disintegration in liquid electrolytes. Since then, solid-state variants have been created to increase stability and efficiency [16]. Charge transport layers in p–i–n and n–i–p planar structures are made of p- and n-type materials, respectively. The p–i–n structure, developed from organic solar cells, provides lower temperature processing, flexibility, and decreased hysteresis than typical mesoporous architectures [17, 18]. Multi-junction cells combine perovskite with other materials to absorb a wider range of wavelengths and collect a broader spectrum of sunlight, especially focused on increasing their efficiency into the infrared region, potentially overcoming the Shockley-Queisser efficiency limit [19]. On flexible substrates, we achieved a validated PCE of 19.5%. These cells are crucial for applications that need lightweight and flexible solar panels [20]. In comparison to 3D perovskites, 2D perovskites have higher stability but poorer efficiency. Hybrid 2D/3D constructions are designed to strike a compromise between efficiency and stability. The additions also help to improve the stability of perovskite solar cells under ambient conditions, addressing an important barrier in the commercialization of these materials [21, 22].

The solar cell consists of multiple materials, some are lead-based and harmful to humans, animals, and the environment, which cause serious health issues and even death, to overcome this, we should move to lead-free material [23]. The environmental and health effects of lead leaking from perovskite solar cells are a concern. Commercialization depends on techniques to lower toxicity and create non-toxic substitutes [24]. A review of lead, tin, bismuth, and organics in developed perovskite photovoltaic technology reveals possible risks to human health and the environment, suggesting toxicity issues with perovskite solar cells [25]. By comparing the lead and non-lead material, lead material produces more efficiency while compared to non-lead material [7]. One major problem that prevents perovskite solar cells from being commercially viable is the toxicity of lead in them. The goal of the research has been to create lead-free substitutes, like bismuth, copper, and tin-based perovskites, which have the potential to be less harmful while yet retaining desired optoelectronic qualities. Furthermore, combined cation and halide perovskites present viable answers to the efficiency and stability problems.

Despite its impressive high efficiency, perovskite solar cells’ low stability remains an important barrier to commercialization [26]. Instability is caused by external as well as internal factors, such as ion migration, oxygen, moisture, light, heat, and molecule dissociation [27, 28]. Moisture causes the perovskite layer to become very unstable, which speeds up deterioration and reduces efficiency [29, 30]. High temperatures and constant light affect perovskite materials instability, which results in breakdown and a shorter operating life [31]. The total stability of the device is significantly impacted by the instability of the perovskite absorber layer and the interfaces between other layers [32]. Even though all-inorganic perovskites have superior compositional and chemical stability than organic–inorganic hybrids, their usage is limited due to phase instability [33]. The stability problems that impact PSCs’ long-term performance are intimately linked to current–voltage hysteresis and ion migration inside the perovskite layer [34]. While certain environmental stability issues may be resolved by modern encapsulation methods, intrinsic factors like thermal stability continue to be challenging [35].

In this [36] study investigates the effects of binary chemicals, 2-phenylethylammonium iodide (PEAI) and ethylenediammonium diiodide (EDAI2), on formamidinium tin iodide (FASnI3) perovskite solar cells (PSCs). While EDAI2 is located on the grain’s margins and acts as a linking agent to enhance the linking between nearby grains, PEAI is crucial for decreasing the number of dimensions of the perovskite particles from 3D to a combination of 2D/3D.The improvements that have been made, especially in perovskite-based TSCs, are thoroughly reviewed in the present article [37], alongside an emphasis on the material’s arrangement, bandgap technology [38, 39], and crystallizing methods used. The invention of various TSC types, such as perovskite/inorganic, perovskite/organic, perovskite/dye-sensitizer, perovskite/perovskite, and perovskite/quantum dots (QDs)-based solar cells, is explained and evaluated in more detail. An effective interior bifacial perovskite photovoltaic system (i-BPPV) that can capture the lightest from both the top and bottom positions has been intended in this work [40]. A visible bottom electrode (ITO) as well as a top electrode (Au/ITO) surrounded by an array of organic–inorganic mixed perovskite material have been employed for the design and manufacture of the i-BPPVs.

As given [41], in order to create a double-cation/double-halide (DCDH) perovskite framework, particular MA_1−x_FA_x_PbI_3−x_Br_x_, tailored for effective inside spite gathering, and have examined the consequences of adding formamidinium bromide (FABr) to methylammonium lead iodide (MAPbI 3) perovskite. According to the simulation of indoor LED light at 1000 lx, the indoor photovoltaics (IPVs) made using the DCDH perovskite layer demonstrated a significantly greater short-circuit current density (J_SC_) of 172.64 μ A·cm^−2^, an open-circuit voltage (V_OC_) of 0.91 V, and a power conversion efficiency (PCE) of 36.29% compared to the MAPbI_3_ IPVs’ J_SC_ of 159.03 μ A·cm^−2^, V_OC_ of 0.84 V, and PCE of 27.89%.The possible application of mixed methyl ammonium/formamidinium cations (MA + /FA +) perovskite materials for capturing energy from indoor LED light sources was investigated in [42]. When FA + is added to methyl ammonium lead iodide (MAPbI3), the structure of the crystal changes from tetragonal to an intermediate quasi-cubic, giving the perovskite a more stable and effective quasi-cubic morphology. When exposed to an indoor LED lamp at 1000 lx, the manufactured photovoltaic devices [43] showed a performance of 34.07% with mixed cations, as opposed to the benchmark MAPbI3 of 28.63%.

## Methodology

**Fig. 1:**
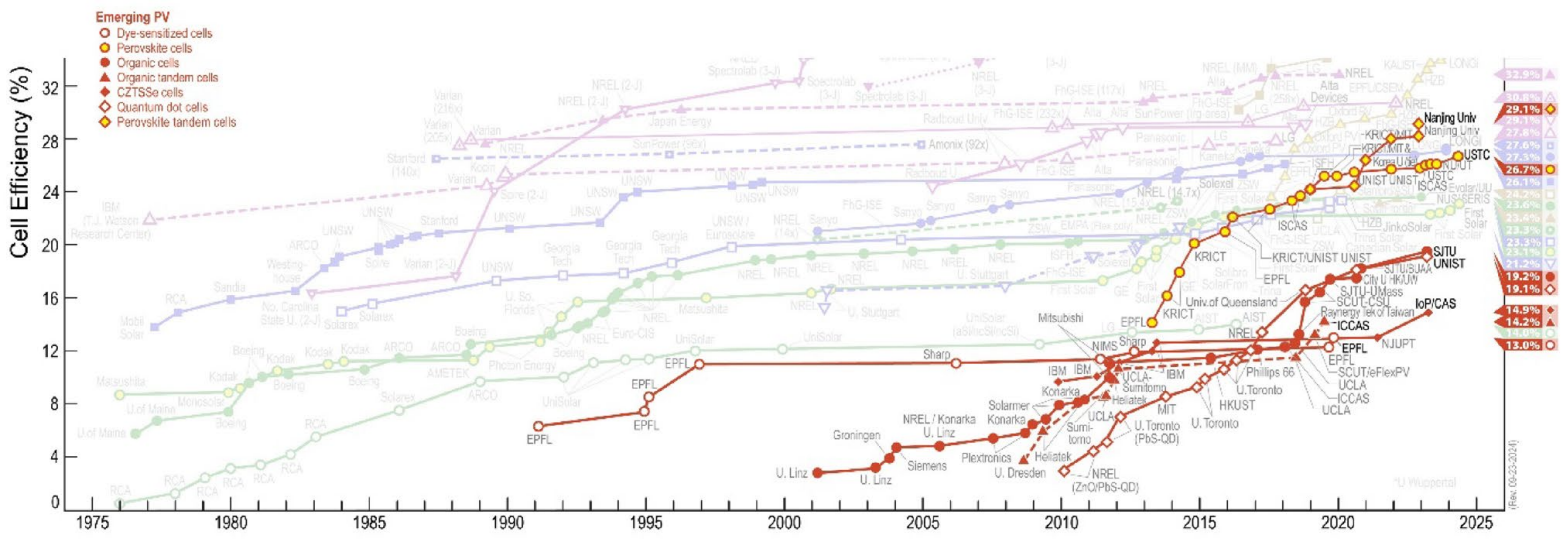
Efficiency graph

The researchers have pointed out that perovskite solar cells function by utilizing the photovoltaic effect. This effect involves light particles (photons) striking the perovskite layer, which then creates energetic electron–hole pairs (excitons). An electron–hole (e–h) pair is created when the electrons are excited and liberated by this energy. The liberated electrons travel towards the hole transport layer. A simple PSC device structure is shown in Fig. 1. Perovskite materials are distinguished by their low exciton binding energy, allowing these excitons to quickly separate into free-moving electrons and holes. These free charges then travel in specific directions: electrons move through a designated layer towards a collecting electrode, while holes travel through another layer in the opposite direction. When electrons reach the conductor, they produce an electric current that can be used to generate electricity. This organized movement of charged particles creates an electrical current, converting light energy into usable electricity [44, 45]. It was emphasized that the efficiency of separating and transporting these charges is critical for the overall performance of perovskite solar cells. This separation delivers an open circuit voltage and a photocurrent [46].

**Fig. 2:**
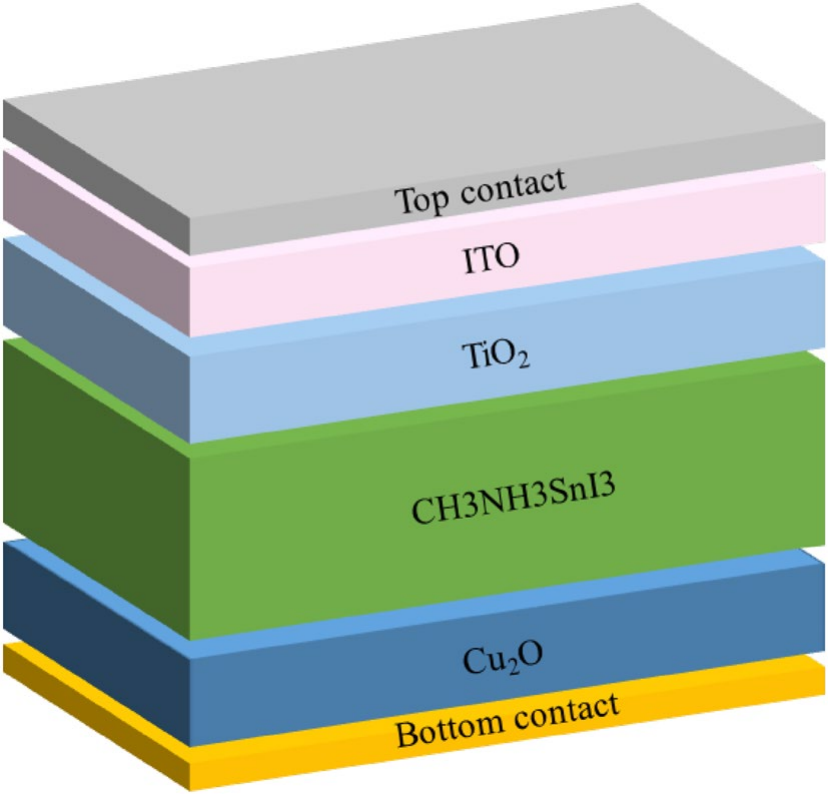
Structure of the proposed solar cell

**Fig. 3:**
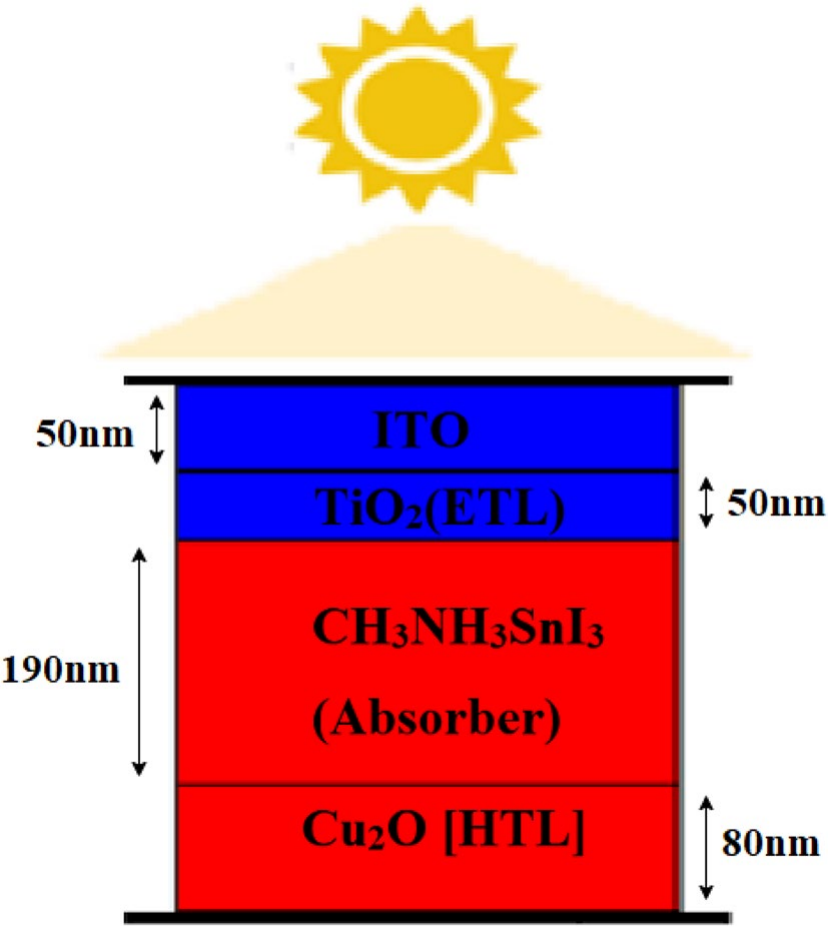
Dimension and layers of the proposed solar cell

The proposed solar cell show in Fig. 2 device is modeled, simulated, and analyzed using SCAPS. The proposed model used in this simulation is shown in Fig. 3, the solar cell is made of p-type and n-type material. This device consists of CH_3_NH_3_SnI_3_, used as an absorber layer, Copper(I) oxide as the hole transport layer, Titanium dioxide as ETL and ITO used as the transparent window layer.

These are the layers used in the proposed work solar cells. The window layer is placed on the top. The sunrays hit the Window layer, which is a transparent layer then the solar rays reach the ETL which is connected to the absorber layer is connected with the HTL, and then finally the ITO is a transparent material that is placed on the top layer where the solar rays are passed through the device. The structure in SCAPS is given below in Fig. 3. Because of its excellent electrical characteristics and compatibility with perovskite materials, TiO_2_ is a widely used ETL in perovskite solar cells. TiO_2_ reduces recombination losses by facilitating efficient electron transport from the perovskite layer to the electrode thanks to its high electron mobility. TiO_2_ and the perovskite material have well-aligned energy levels, which ensures effective charge extraction and reduces energy losses. TiO_2_ helps solar cells last longer since it is chemically stable and resistant to the environmental factors that PSCs are usually subjected to. Because TiO_2_ is so transparent to visible light, the perovskite layer receives the most light possible, which is essential for high efficiency. In PSCs, cuprous oxide is becoming steadily recognized as a potential HTL. Cu_2_O is a p-type semiconductor with a bandgap of around 2.1 eV, making it favourable for hole transport. It is inexpensive, plentiful, and non-toxic. This enables it to be scaled for large-area applications and compatible with flexible substrates. In contrast to organic HTLs, which may deteriorate with time, inorganic HTLs, such as CuO, provide superior thermal [47] and chemical durability. The two contacts that make up solar cells are the front and rear contacts, which serve as the anode and cathode, respectively. One of these contacts is positive, while the other is negative. The charges generated by the cell are collected via these contacts [48]. To improve light penetration into the cell, the top contact is often transparent, like FTO and ITO.

To effectively let light into the perovskite layer, ITO offers remarkable optical transparency in the visible spectrum [49, 50]. ITO effectively transports charges from the perovskite layer to the external circuit because of its high electrical conductivity. ITO has a wide range of application options since it may be deposited on many substrates, such as glass and flexible materials [51]. ITO provides a suitable interface for electron extraction, helping to minimize recombination losses and enhance device efficiency. Gold is used as a back metal contact. This software may simulate a wide range of properties, including mathematical models and material properties. This software allows the user to use various types of solar spectrums like AM1.5G, AM0, AM1.5Gedition2, and AM1.5D. We can also test our device under various temperatures and with different resistances. We can use a maximum of seven different types of material or layers in a single cell. This software enables us to carry out current-to-voltage, capacitance-to-voltage, capacitance-to-frequency, and quantum efficiency analyses.

The electron transport layer’s primary purpose is to make electron-selective contact with the perovskite light-absorbing layer, increasing the efficiency of auto-generated electron extraction. It reduces the recombination of the electron holes and stops the hole from moving to the counter electrode [52]. The electron transport layer enables the transmission of an electron from one region to another. To facilitate the separation of electrons and hole pairs, holes are collected and transported from the perovskite light-absorbing layer by use of the hole transport layer. High hole mobility, wide band gap, straightforward solvent treatment technique, and high film forming ability are all necessary properties when choosing materials for the HTL.

The numerical parameters used in our device consist of CH_3_NH_3_SnI_3_, which is a lead-free perovskite substance that is utilized as an absorber layer. It has parameters like bandgap, electron affinity relative permittivity [53]. Cu_2_O is used as a HTL [54] and ITO is used as a window layer [55]. On comparing ETL materials like IGZO, C_60_, SnO_2_, ZnO, and TiO_2_, by simulation, we came to know that TiO_2_ has better performance when compared to others. This shows that TiO_2_ can be used as an ETL layer [56]. Table 1 lists the parameter values. The optimized values are listed below.

**Table 1:** Material parameters of the proposed solar cell

Parameter	ITO	TiO_2_ [ETL]	CH_3_NH_3_SnI_3_ (absorber)	Cu_2_O [HTL]
Thickness (nm)	50	50	190	80
Bandgap (eV)	3.50	3.20	1.30	2.170
χ (eV)	4.00	4.260	4.170	3.20
Εr	10	9	8.20	7.11
Nc (cm^−3^)	2e18	2e18	1e18	2e17
Nv(cm^−3^)	1.8E 19	1.8E 19	1E 18	1.1e19
µn/(cm^2^/V·s)	2e01	2e01	1.6e0	2e02
µMaterial properties p/(cm^2^/V·s)	1e01	1e01	1.6e0	8e01
NA (1/cm^3^)	2e19	1e16	0e0	0e0
ND (1/cm^3^)	0e0	0e0	3.2e10	1e15

In this device, Band-band recombination is used. The Band-to-band recombination is the radiation-driven transition of the electrons from the conduction band to the valence band. The defect density is added for all the HTL, window, ETL, and absorber layers. Energetic distribution is single for all the layers except for the absorber layer, which is Gaussian. The defect type is accepter for p-type and donor for n-type. Capture cross-section electrons (cm^2^) is set to 1e−15 for all the layers and capture cross-section holes (cm^2^) is also set to 1e−15. Regarding the materials, the values of the Auger electron capture coefficient, Auger hole capture coefficient, and radiative recombination coefficient are defined. The Absorption coefficient is used from the material library which has the spectrum response for a particular wavelength. Some defects and material interfacesare added to the device which are listed below in Table 2.

**Table 2:** Parameters defect density

Parameter	TiO_2_ [ETL]	CH_3_NH_3_SnI_3_ (Absorber)	Cu_2_O [HTL]
Defect type	Neutral	Neutral	Neutral
Capture cross section for electrons (cm^2^)	1.00E−15	1.00E−15	1.00E−15
Capture cross section holes (cm^2^)	1.00E−15	1.00E−15	1.00E−15
Energetic distribution	Single	Gaussian	Single
Energy level with respect to eV (above eV, eV)	6.00E−01	6.00E−01	6.00E−01
Characteristic energy (eV)	–	1.00E−01	–
Nt total (1/cm^3^)	1.00E+15	1.00E+10	1.00E+14
Nt total (1/eV/cm^3^)	–	5.64E+10	–

The effective density of states for valence band holes (Nv) and conduction band electrons (Nc) at room temperature (300 K) can be determined by applying the following Eqs. (1) and (2).

1 $$\text{Nc}=2{\left[\frac{2\pi {m}_{e}KT}{{h}^{2}}\right]}^{3/2}$$

2 $${\text{Nv}=2\left[\frac{2\pi {m}_{h}KT}{{h}^{2}}\right]}^{3/2}$$

Furthermore, the intrinsic carrier density of the states can be determined by applying formula (3).

3 $$\text{ni}=\sqrt{Nc{Nve}^{-\left(EC-Ev\right)/2KT}}$$

where Ec−Ev = Eg, which stands for the energy band gap, and K for Boltzmann constant (1.3806 1023 J K1), T for absolute temperature (usually 300 K), h for the Planck constant (6.626 10–34 m^2^ kg/s), and me and mh for the effective masses of both electrons–holes respectively [57]**.**

## Results and discussion

The proposed device is simulated and the results are obtained through SCAPS using the previously mentioned parameter and the obtained response is explained below. By simulating the device which has parameters in ETL exhibits output which has a thickness of 30 nm, electron affinity is 4.26 eV and bandgap of 3.2 eV, Absorber has thickness of 110 nm, the band gap is of 1.3 eV and electron affinity is 4.17 eV, HTL has a thickness of 50 nm, the bandgap is of 2.17 eV and electron affinity is 3.2 eV and obtain output V_OC_ value of 1.5731 V, J_SC_ value of 20.152 mA/cm^2^, FF value of 82.18%, and efficiency of 26.06%. The V_OC_ is the maximum voltage of this device. It does not depend on the intensity of light but it depends on the structure of the device. If the size of the device is increased the open circuit voltage will increase because it could accumulate more charges so that it can hold up more energy. J_SC_ is the maximum current that which device can obtain. This short-circuit current depends on the intensity of the light. This device works under the series resistance of 1 Ohm cm^2^ and shunt resistance of 100 Ohm cm^2^ under the temperature of 300 K. The spectrum used in this simulation is AM1_5G 1 sun. The device is further optimized.

### Effect on CH_3_NH_3_SnI_3_ layer thickness

**Fig. 4:**
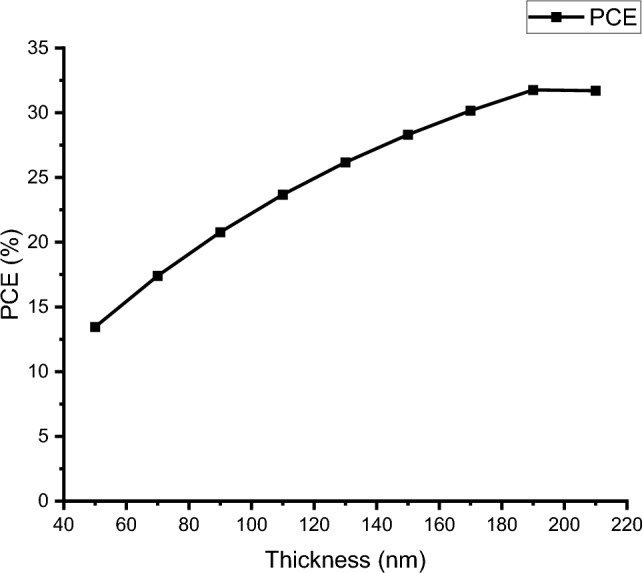
Impact on the thickness of the active layer

In a PSC, the active layer is fundamental. The thickness of the active layer has an impact on the whole cell’s efficiency. Generation and the conduction of electron and hole pairs are both performed by this layer. When the thickness of an active layer is high, it can absorb more photons from the sun’s rays. If the thickness of the perovskite layer is very large at a certain stage it makes a large gap between ETL and HTL so that efficiency begins to decrease. So, the thickness of the perovskite layer should be in optimal thickness. Table 3 lists the impact on the active layer’s size, which is from 50 to 210 nm, and the efficiency to thickness is shown in graphical format in Fig. 4. The thickness of the ETL and HTL layers are also optimized.

**Table 3:** Effect on the active layer’s thickness

Active layer thickness in nm	Efficiency (%)	V_oc_ (V)	J_SC_ (mA/cm^2^)	FF (%)
50	13.45	1.584	11.92	71.15
70	17.40	1.581	14.53	75.71
90	20.78	1.578	16.80	78.35
110	23.68	1.574	18.79	80.01
130	26.17	1.571	20.53	81.11
150	28.31	1.3089	22.05	81.85
170	30.16	1.5652	23.387	82.38
190	31.73	1.3089	29.780	81.40
210	31.70	1.560	25.10	82.48

**Fig. 5:**
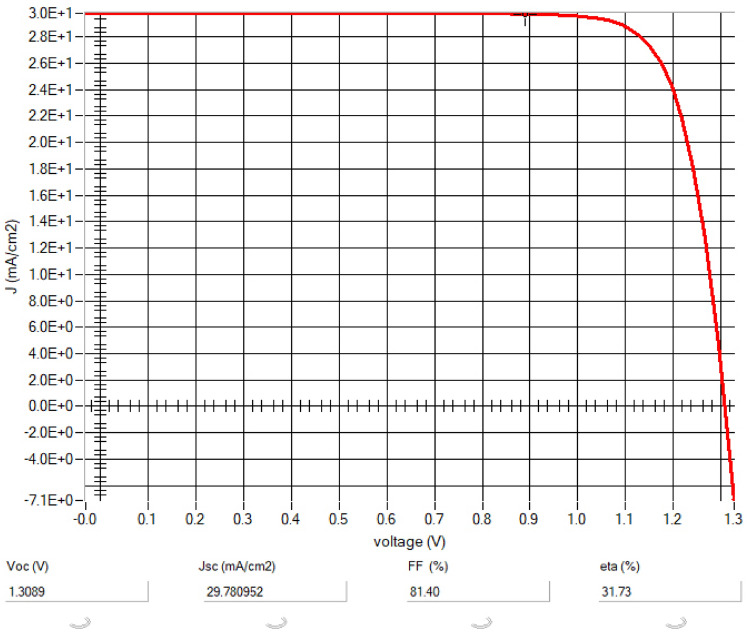
IV characteristics of the proposed solar cell

The performance of the device is increased by optimizing the values of thickness, bandgap, electron affinity, and other parameters the device is optimized and the output of the device is improved by 15.7% under the series resistance of 1 Ohm cm^2^ and shunt resistance of 100 Ohm cm^2^ under the temperature of 300 K and tabulated in Table 4 and current to voltage curve have been shown in Fig. 5.

**Table 4:** Device output parameters

PCE	V_OC_	J_SC_	FF
31.73	1.3089	29.780	81.40

**Fig. 6:**
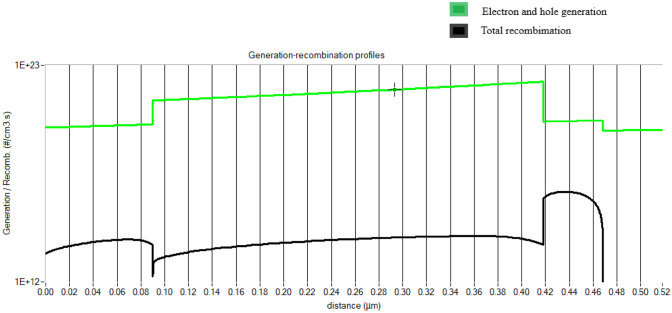
Electron–hole generation and total recombination

**Fig. 7:**
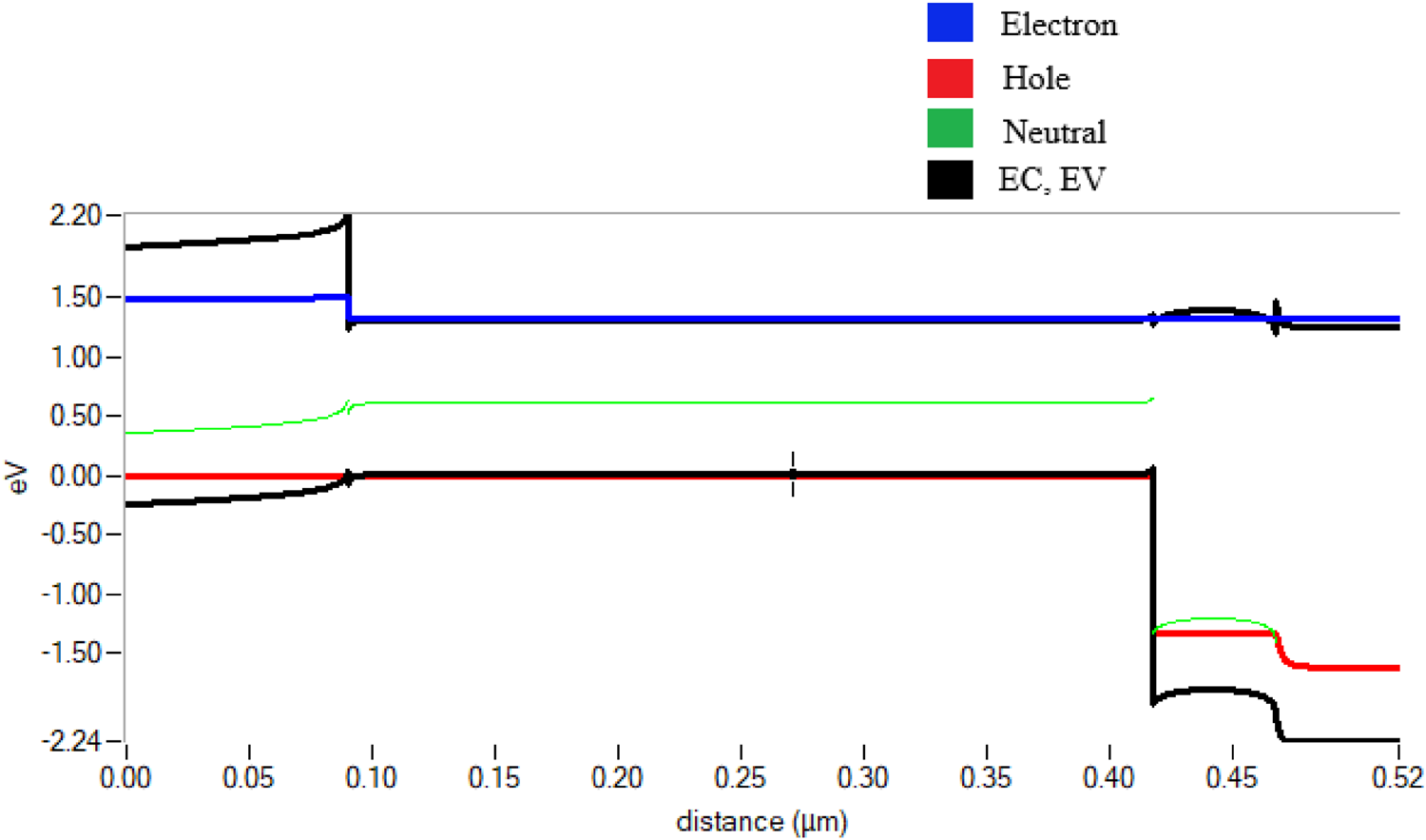
Energy band diagram

Recombination current is that the current results from the recombination of electrons and holes. Carriers (electrons and holes) should be created within a diffusion distance from the connection. Carriers need to be created closer to the junction than they are to the recombination site for localized high recombination sites (such as unpassivated surfaces or grain boundaries). Differing energy photons have differing chances of collecting because of specific recombination sites at both the front and back surfaces. The effect of recombination on light-generating current is quantified by quantum efficiency. Carriers produced by infrared light are mostly affected by high rear area recombination. The diffusion current in a forward-biased PN junction is affected by recombination. Higher diffusion current from increased recombination lowers the V_oc_. The recombination current of the proposed device is shown in Fig. 6. It clearly shows the total generation and recombination of the charge carrier in graphical format which also includes SRH, radioactive, and auger recombination. Figure 7 shows the energy band diagram.

**Fig. 8:**
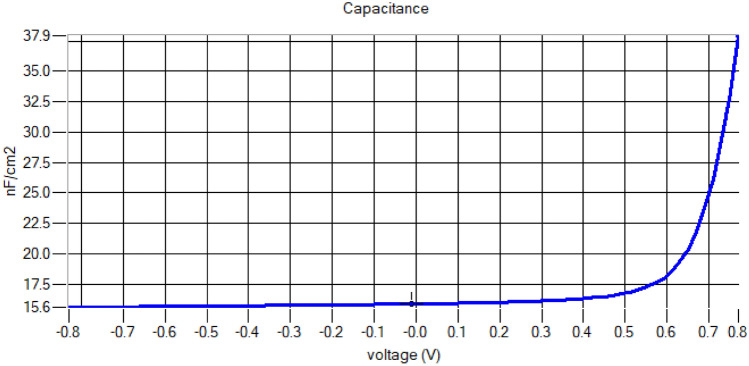
Capacitance–voltage curve

There will be an increase in capacitance value between every layer of the solar cell which the increase the capacitance voltage. Also because of this effect, the maximum capacitance obtained is 0.8 V. By analyzing Fig. 8, one can see a steady increase in capacitance and there is a sudden rise in capacitance value. From 0 to 0.6 V there is a slight change in capacitance value but after 0.6 V there is a drastic increase in the capacitance. We can reach a maximum capacitance of up to 38.4 nF/cm^2^.

### Temperature analysis

**Fig. 9:**
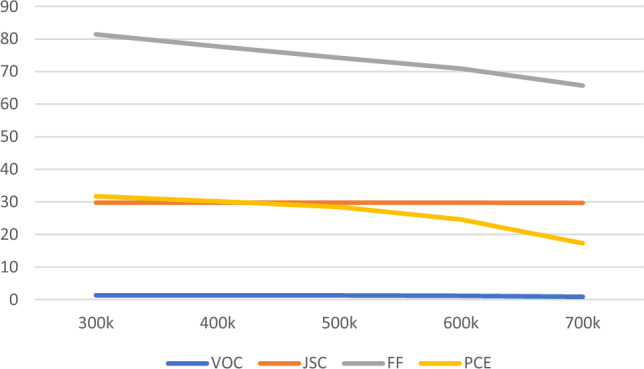
Impact of solar cell due to temperature

It’s common for a device to increase in temperature. If temperature increases,the performance of a device willdecrease. A solar cell is directly placed under the sunlight so there will be an increase in temperature. The temperature analysis has been done and tabulated in Table 5. By considering all the below Fig. 9 with various temperatures came to know that while increasing the temperature the efficiency keeps on decreasing. In 300–600 k, we can see a slight change in efficiency but compared to 600 k there is a drastic drop in efficiency. The proposed device works efficiently in 300–600 k.

**Table 5:** Impact of solar cell due to temperature and its stability

Temperature (k)	V_OC_	J_SC_	FF	PCE
300	1.3089	29.780	81.40	31.73
400	1.303	29.78	77.69	30.17
500	1.28	29.78	74.23	28.44
600	1.16	29.78	70.90	24.61
700	0.88	29.7	65.7	17.29

### Quantum efficiency analysis

The efficiency with which a solar cell converts incoming photons into electrical charge carriers (electrons or holes) is known as its quantum efficiency (QE). It is the ratio of the number of charge carriers that the solar cell collects to the number of photons that strike the cell with a certain energy. The incident light’s wavelength might affect the QE. A solar cell with a high QE at a given wavelength is very effective at converting photons at that wavelength into electrical current. On the other hand, inefficiencies, often brought on by recombination losses or inadequate absorption, are indicated by a low QE. The proposed solar cell has a high QE in the 300–500 nm wavelength range and obtains an outstanding QE.

**Fig. 10:**
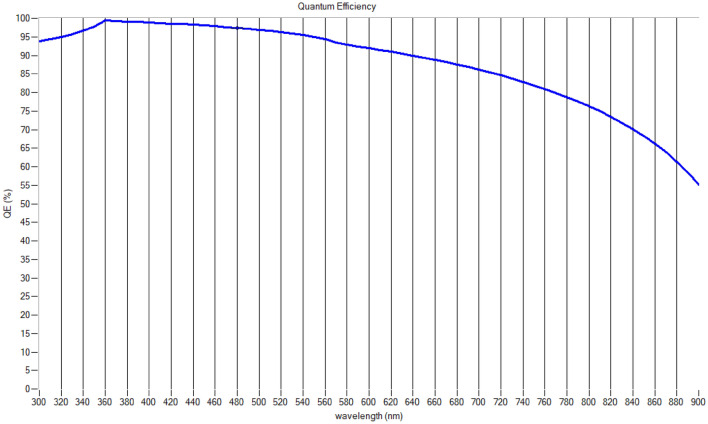
Understanding the cell’s quantum efficiency over a range of wavelengths

Series and shunt resistances have a substantial impact on solar cell performance, affecting voltage, current, and efficiency. Series resistance (Rs) lowers overall efficiency by decreasing fill factor, open-circuit voltage, and short-circuit current [58]. Conversely, shunt resistance (Rp) is essential for reducing current mismatches, particularly in solar cells linked in series, which improves power production and durability under a range of lighting situations (Fig. 10).

Compared to other similar work, this device performs well and has better efficiency. This device has a fairly high PCE when compared with other lead-free PSCs. When comparing CsSnI_3_-based perovskite [58, 59] have better efficiency and Jsc, this is due to the size of the device. This CsSnI_3_-based device has a difference of 80% in size when compared to the proposed device. When comparing the proposed device with similar CH_3_NH_3_SnI_3_-based CH_3_NH_3_SnI_3_ [60] it has HTL and ETL of n-CH_3_NH_3_SnCI_3_/p-CH_3_NH_3_SnI_3_ and has an efficiency of 24.81, The proposed design performs 28% better than the existing device. By comparing with other devices this proposed device has comparatively high efficiency. This comparison shows how the proposed device performs better than other existing work and the comparison has been tabulated in Table 6.

**Table 6:** Performance comparison

References	Perovskite type	Jsc (mA/cm^2^)	Voc (V)	FF (%)	PCE (%)
[60]	CH_3_NH_3_SnI_3_	28.11	1.00	87.69	24.81
[61]	MAPb1−xEuxI_3_	20.8	1.02	74.2	15.7
[62]	Cs_2_GeSnCl_6_	26.35	1.02	73	16.35
[63]	Cs2TiBr6	45.4	0.515	79.17	18.53
[64]	Cs_2_AgBiBr_6_	21.35	1.501	82.87	26.89
[65]	Cs_2_AgBi_0.75_Sb_0.25_Br_6_	18.6	1.76	92.37	30.3
[66]	Csx(FA_0.4_MA_0.6_)_1−x_PbI_2.8_Br_0.2_(3D)-(PDA)(MA)_n−1_PbnI_3n+1_	22.08	1.50	86.27	28.60
Proposed device	CH_3_NH_3_SnI_3_	29.780	1.3089	81.40	31.73

## Conclusion

In summary, a comparative analysis of existing lead-free PSCs underscored the superior performance and environmental sustainability of the proposed device. Investigation delves into pioneering the design and virtual emulation of an environmentally friendly perovskite solar cell, featuring CH_3_NH_3_SnI_3_ as the light-absorbing layer, Copper(I) oxide as HTL, Titanium dioxide as the electron transport layer, and Indium tin oxide as the transparent window stratum. Leveraging the sophisticated SCAPS-1D simulation framework, we meticulously fine-tuned the device parameters, culminating in a stellar PCE of 31.73%. This remarkable feat was accompanied by a substantial J_sc_ of 29.780 mA/cm^2^, an impressive FF of 81.40%, and a V_oc_ of 1.3089 V. Furthermore, our inquiry scrutinized the repercussions by varying thickness of the active layer, unveiling optimum dimensions that markedly bolstered the efficacy of the device. Through temperature analysis spanning 300–500 K, we gleaned insights into the performance of the device under diverse thermal conditions, underscoring its resilience within this temperature range. Despite the challenges inherent in lead-free materials, our study showcases the viability of harnessing non-toxic perovskite compounds to achieve unparalleled solar energy conversion efficiency. With further optimization and empirical validation, these findings hold the promise of catalyzing the widespread adoption of sustainable energy alternatives, thereby mitigating the adverse environmental impacts of conventional energy sources.

## Data Availability

The data samples have been reasonably requested.

## code availability

The relevant code with the manuscript is also available and would be available, if will be asked to do so later.
